# C-753-03. Intravenous Tenecteplase for the Treatment of Severe Frostbite

**DOI:** 10.1093/jbcr/irag033.024

**Published:** 2026-04-06

**Authors:** Sarah Micucci, Kathryn E Smith, Rosemary E Paine, Damien W Carter

**Affiliations:** University of New England, Standish, ME; Maine Medical Center, Portland, ME; MaineHealth Medical Center, Portland, ME; MaineHealth Medical Center, Portland, ME

## Abstract

**Introduction:**

Intravenous (IV) and intra-arterial (IA) thrombolytics are recommended for treatment of severe frostbite to reduce amputation rates and level of amputation needed. A recent systematic review described an average limb salvage rate of 78.7% with thrombolysis. Most trials examining thrombolytics utilized alteplase (tPA). One study reported the use of IA Tenecteplase (TNK), but no studies have examined its systemic use for frostbite. IV TNK’s ease of dosing makes it an attractive treatment option, especially given time to reperfusion is crucial to limb salvage. We describe our institutional experience with the novel use of IV TNK for severe frostbite.

**Methods:**

A retrospective chart review of patients >18 years of age with severe frostbite admitted to the burn service at a level 1 trauma center and received IV TNK from December 2022 to February 2025 was conducted. Effectiveness of TNK was determined by need for amputation and number of digits amputated. Safety was determined by number of bleeding complications. Tissue perfusion on indocyanine green (IcG) microangiography before and after TNK administration was also collected.

**Results:**

Six males with median age 32 (range 27 - >89) years and grade 4 frostbite injuries of lower extremities (n = 5) or upper extremities (n = 1). At the time of injury, 83% were unhoused or experiencing housing instability, 83% had a mental health disorder diagnosis, 33% had a positive blood ethanol level, and 33% tested positive for >1 illicit substance. The median hospital stay was 9 (2-34) days. TNK dose was 0.25 mg/kg (16-23.5 mg), with median time to administration 7.2 (4-17) hours from presentation. Prior to administration, 100% had decreased perfusion to the affected region on IcG imaging. 83% underwent IcG imaging after administration. There was a limb salvage rate of 17 of 41 (41%) affected digits, with the remaining digits requiring amputation. Minor bleeding occurred in 1 patient and major bleeding (retroperitoneal hematoma) occurred in 1 patient of advanced age (>89 years) and current use of apixaban.

**Conclusions:**

Our case series is the first to describe IV TNK administration for management of severe frostbite. Our results suggest TNK increases tissue perfusion on IcG imaging and is safe for otherwise healthy patients but requires comparison to standard of care. In patients of advanced age or using anticoagulants, a careful risk assessment should be conducted. Further studies are needed to investigate the limb salvage rates and safety of IV TNK compared to tPA.

**Applicability of Research to Practice:**

Frostbite injury and amputation contribute to significant disability and disproportionately affect unhoused and mentally ill populations. Determining the optimum management of frostbite to decrease amputation and complication rates is at the forefront of burn practice and research. Use of IV TNK offers ease of administration as compared to TPA (1 dose and shorter infusion time), which may decrease time to thrombolysis and increase limb salvage.

**Funding for the Study:**

N/A.

